# The role of dynamic phenotypes in cancer

**DOI:** 10.18632/oncotarget.28006

**Published:** 2021-09-14

**Authors:** Luana S. Lenz, Guido Lenz

**Affiliations:** ^1^Departamento de Biofísica, Universidade Federal do Rio Grande do Sul, Porto Alegre, RS, Brazil; ^2^Centro de Biotecnologia, Universidade Federal do Rio Grande do Sul, Porto Alegre, RS, Brazil

**Keywords:** dynamic phenotype, fitness, tumor resistance, tumor evolution, single cell

## Abstract

The question of whether cancer recurrence is mediated by a process that is exclusively Darwinian or that involves both Darwinian and Lamarckian processes is long standing and far from answered. The major open question is the origin of variation, whether it relays exclusively on stable, mostly genetic, mechanisms or whether it can also involve dynamic processes. Recent evidence with single-cell epigenomic and transcriptomic profiling and measurement of phenotypes in colonies indicate that several phenotypes quickly change with a few cell divisions. Most importantly, cell fitness under basal as well as in the presence of chemotherapeutic agents changes considerably over short periods of time and this dynamic is reduced by epigenetic modulators. These studies contribute to establish the dynamic nature of fitness and are key for the interplay between cancer cell dynamics and stable genetic and epigenetic alterations in the survival of a few cancer cells after therapy.

## INTRODUCTION

A major challenge to overcome in cancer is to understand how cells adapt to survive to therapy. The classical Darwinian mechanism of resistance, in which random mutations permit natural selection, plays a role in cancer resistance, but is far from the only process involved. Mechanisms of tolerance generated through dynamics in cellular processes can also support therapy evasion. Therefore, the increased knowledge about the dynamics in several phenotypes in cancer cells in the last years has opened our eyes on the degree of these dynamics and their potential role in therapeutic resistance [1, 2]. Such fluctuation together with the occurrence of different level of expression memory along generations [3, 4] can contribute to heterogeneity found among related cells, and indicate that the heterogeneity of a tumor goes far beyond mutations.

So far, most studies of dynamics have focused on signaling pathways, expression of specific genes and modulation of cellular processes [5, 6], however, the most important omniphenotype in cancer is the number of live descendant of a cancer cell after a defined time, i.e., its fitness. Several studies have shown the improve on cell fitness due to phenotypic changes [7, 8], however the focus is usually populational and not on the dynamics of single cells. A very remarkable example is the impact of the flower isoforms on fitness. This transmembrane protein has four isoforms, two of them are related with a winner phenotype and the other two with a loser phenotype, thus being considered a ‘fitness fingerprint’ [9, 10]. The outcome depends on the expression of neighboring cells and other proteins involved in signaling [9, 11, 12]. Even though it is known that the flower protein expression is important to tumor growth and metastasis [9, 13], it is not known if the expression of the flower gene is stable or fluctuates over time in single cells. In this regard, the question remains whether a winner cell will produce descendants that are all winners, or will the formed clone be a mix of winner and looser cells in a similar proportion of the population of non-related cells?

**Figure 1:**
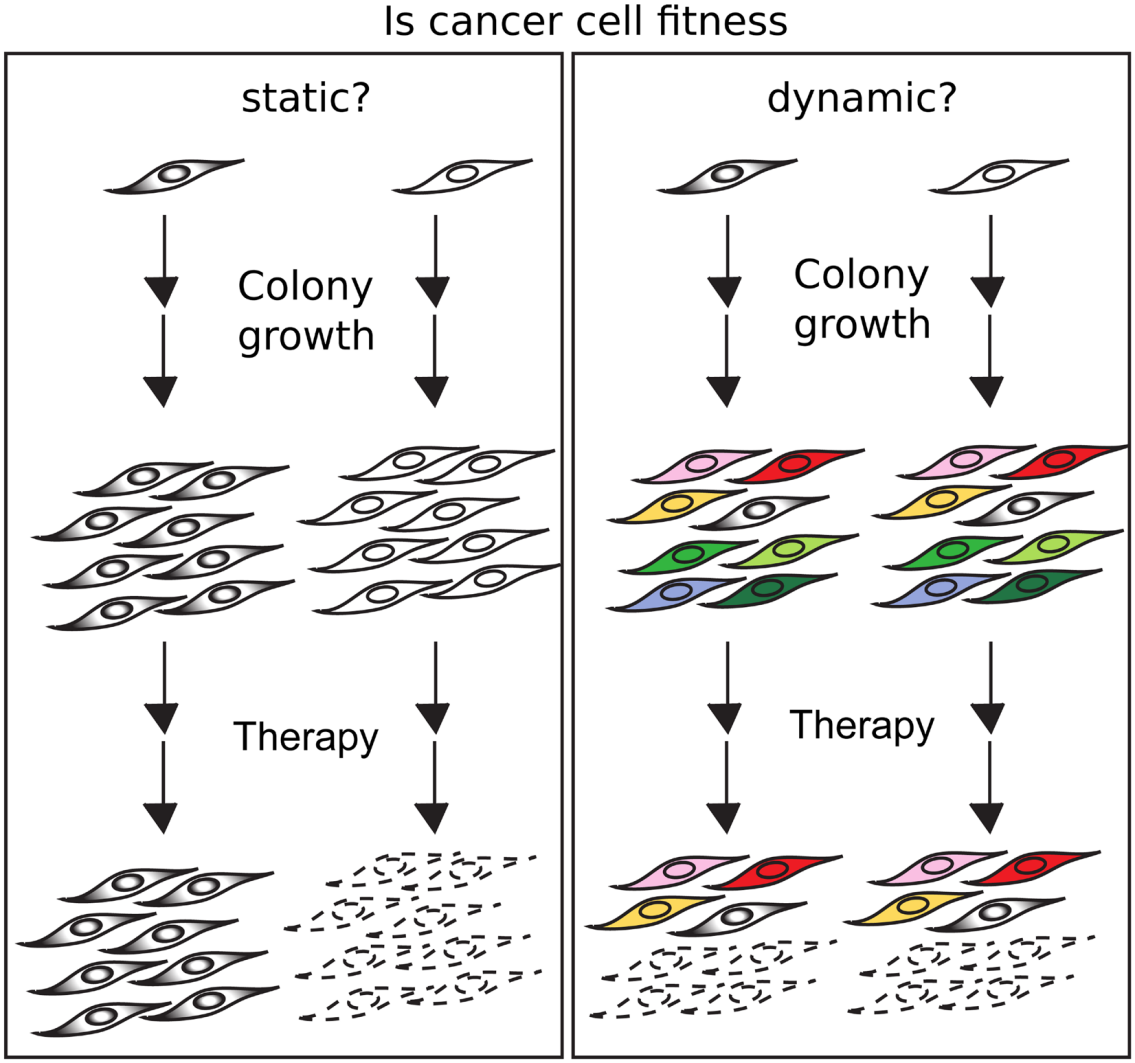
If fitness is conserved, cells with high and low fitness will generate colonies which will be resistant or sensitive to a therapeutic agent (left). If fitness changes with time and/or division, the colonies formed from high and low fitness cells will be similar, in which both colonies will undergo fractional killings in the presence of a therapeutic agent (right).

The major challenge to measure the dynamic of fitness at single cells is that when a cell dies, the ability to get information about its future is lost, and when a cell divides, the cell is not the same anymore. To overcome this methodological barrier and address the dynamics in cancer cell fitness, we created the Dynamic Fitness Analysis (DynaFit) method [14]. The rationale of DynaFit is that if fitness does not change during the formation of colonies, the fitness level of the founding cell will be phenotocopied to all cells in the colony and therefore the comparison between colonies will reveal a variance in fitness similar to the variance of their founding cells. However, if fitness changes over time or with cell division, cells with diverse fitness levels will produce colonies that will be formed from high and low fitness cells and therefore these colonies will have a behavior much closer to the average fitness and therefore a variance in fitness among colonies much lower than the variance of their founding cells (Figure 1). As DynaFit takes into account the size of the colony, one can estimate the degree of dynamics in fitness.

The results obtained with DynaFit clearly show that normal and cancer cells change their fitness after only a few generations both under normal growth condition or in the presence of chemotherapeutic agents [14]. This indicates that tolerance to this agents fluctuates and may thus contribute for the high rate of failure of treatment of tumors such as glioblastoma.

The origins of phenotypic fluctuations are diverse and far from understood. We observed that sister cells already have different levels of MAPK signaling levels and that cell cycle desynchronization and the induction of apoptosis and senescence becomes different among cells in colonies after a few divisions [14]. Others have also shown the contribution of cell cycle-dependent fluctuations [15], transcriptional noise [16, 17] and epigenetic modulation [18] on the variation of cells. Tolerance is a dynamic phenomenon caused by epigenetic reprograming [19, 20] which can either remain dynamic or be fixed in population through genetic assimilation [21] or genetic resistance [22]. We hypothesized that alterations in epigenetic pathways through epigenetic inhibitors could alter the dynamic of fitness. Indeed, cells treated with both histone deacetylase inhibitor and DNA methyltransferase inhibitor maintain a variance among colonies similar to the observed in single cells. Besides that, fractional killing was reduced in bigger colonies when they were treated with epigenetic inhibitors along with TMZ compared with colonies treated only with TMZ, indicating that when epigenetic mechanisms are blocked the sensitive phenotype is phenocopied through generations [14].

The strategy to freeze the level of tolerance of cells has the drawback of fixing both the good and the bad. The strategy of evolutionary trap could be employed to deal with cells with a stabilized tolerant state to a given drug [23]. In that case, the pressure of a drug will positively select a trail that confers an advantage, but also produce fitness trade-offs, that can be explored with a second therapeutic intervention. This concept has been demonstrated in relation to the aneuploid phenotype [24] and the antagonistic pleotropy between bromodomain and BCL-2 inhibitors in acute myeloid leukemia [25]. However, the practical applicability of the rationale of evolutionary trap in a therapeutic setting is still in the distant future.

The incorporation of dynamic processes in our knowledge of cancer biology is fundamental for basic and applied cancer research. Integrating Darwinian and Lamarckian evolutionary concepts in cancer cell adaptation to therapeutic pressure is key for moving forward in the comprehension on how cancer cells adapt to the challenges of therapy and is the only way to improve therapies available.
